# How Should Global Fund Use Value-for-Money Information to Sustain its Investments in Graduating Countries?

**DOI:** 10.15171/ijhpm.2017.25

**Published:** 2017-02-27

**Authors:** Kitti Kanpirom, Alia Cynthia G. Luz, , Kalipso Chalkidou, Yot Teerawattananon

**Affiliations:** ^1^ Bureau of Health Administration, Ministry of Public Health, Nonthaburi, Thailand.; ^2^ HITAP International Unit, Ministry of Public Health, Nonthaburi, Thailand.; ^3^ Global Health and Development Team, Imperial College London, London, UK.; ^4^ Health Intervention and Technology Assessment Program (HITAP), Ministry of Public Health, Nonthaburi, Thailand.

**Keywords:** Value-for-Money (VFM), Global Fund (GF), Vertical Programs, Priority Setting, Government Healthcare Investments

## Abstract

It has been debated whether the Global Fund (GF), which is supporting the implementation of programs on the prevention and control of HIV/AIDS, tuberculosis (TB) and malaria, should consider the value-for-money (VFM) for programs/interventions that they are supporting. In this paper, we critically analyze the uses of economic information for GF programs, not only to ensure accountability to their donors but also to support country governments in continuing investment in cost-effective interventions initiated by the GF despite the discontinuation of financial support after graduation. We demonstrate that VFM is not a static property of interventions and may depend on program start-up cost, economies of scales, the improvement of effectiveness and efficiency of providers once the program develops, and acceptance and adherence of the target population. Interventions that are cost-ineffective in the beginning may become cost-effective in later stages. We consider recent GF commitments towards value for money and recommend that the GF supports interventions with proven cost-effectiveness from program initiation as well as interventions that may be cost-effective afterwards. Thus, the GF and country governments should establish mechanisms to monitor cost-effectiveness of interventions invested over time.

## Introduction

The Global Fund (GF) is one of the biggest worldwide health development initiatives focusing on bringing the end of HIV/AIDS, tuberculosis (TB), and malaria. Since its establishment in 2002, the Fund has relied on financial contributions from both public and private sectors.^1^ In 2015, the GF had received in total US$33 billion in cumulative and fully paid contributions. A pledging conference, during which donors and funders commit to assist the goals of the GF, was held in 2016. This is a timely paper that addresses a long debate in global communities on whether the GF, which implements vertical programs in three major diseases, should consider the value-for-money (VFM) of their investments on which level of governance and how this can be operationalized. The case study of Thailand is presented given the authors first-hand experience in Thailand. Moreover, Thailand is now graduating from GF support and there are discussions on the sustainability of the GF investment. The authors reviewed relevant documents, both published and grey literature, as well as using direct experience of conducting health technology assessments (HTAs)^ [1]^ and health system and policy research to support decision-making in Thailand. We conclude by proposing a conceptual framework that can be used to guide the GF and country recipients in incorporating VFM information for implementing and monitoring the GF programs even well after graduation or transition from support.

## Thailand and the Global Fund

Thailand is an upper-middle income country with a population of 67 million and an average income per capita of $5561 in 2014.^2^ Since 2002, Thais have enjoyed universal health coverage (UHC) funded through public sources. Seventy-five percent of the total health expenditure in the country, amounting to 513 billion baht or equivalent to $16 billion, is government funded.^3^ Although Thailand is facing the burden of non-communicable diseases (NCDs) due to its aging society, it still has challenges to overcome, namely communicable diseases, especially HIV/AIDS, TB, and malaria. HIV dropped from the top disease burden among Thais in 1999 with 1.3 million disability-adjusted life years (DALYs) lost, to the fifth place in 2009 with 0.44 million DALYs lost. On the other hand, TB is the 17th, with DALYs lost dropping from 0.16 in 1999 to 0.14 million in 2009. Malaria is not a common disease for most Thai communities, except in the border areas. The estimated burden of malaria in 2009 is 0.003 million DALYs lost and is ranked 67th in terms of disease burden.^4^ The UHC benefit package has included TB and malaria treatments since its inception, whereas antiretroviral treatment for HIV was only included in 2005 after the scaling up of treatment efforts from 2002.^5^ The spending for HIV/AIDS accounted for 2.4% of total health expenditure in Thailand or at approximately $330 million in 2011, of which around 80% come from public funding and 20% from others, predominantly the GF.^6^ For TB, the total expenditure is approximately $30 million annually, with the government spending around $20 million. For malaria, the total spending is $9 million, which is mostly from the GF, with the government spending less than $1 million on efforts for the disease.^7^

As the only major overseas development partner who is funding healthcare in Thailand, the GF cumulatively contributed $540 million of grants for prevention and control of HIV/AIDS, TB and malaria since 2003. Because Thailand’s economy is consistently growing, the country is graduating from GF support in 2016 for HIV and TB and in 2017 for malaria. Although the percentage of financial support for HIV and TB from GF is relatively small compared to the budget (less than 10%) that the Thai government currently invests for the national HIV and TB programs,^8^ the target organizations for the GF grants are typically different from those which receive government investment. GF grants target non-state actors (NSAs) and/or community organizations to provide outreach services to populations that are overlooked by or are difficult to reach for the government programs. This is also due to the illegal status of most at-risk populations such as injecting drug users (IDU), female sex workers (FSW), and migrant workers. The formal Thai healthcare sector provides a primarily supportive role for NSAs to assist the GF program for HIV and TB, such as trainings or laboratory support. Because of its emphasis on high priority diseases and conditions by disease burden, UHC may disregard important disease burdens in small populations. However, UHC also focuses on infectious diseases that may begin with minority populations which is justifiable to minimize the possibility of epidemic outbreaks. For malaria, both the informal and formal sectors under various NSAs and the government benefit from the GF grant. The GF grant has been used for training of health professionals and finding cases in formal sectors; meanwhile, the informal sector receives the grant for communication and engagement in broader areas.

Thailand is considered a role model for having achieved UHC for its constituents as well as using evidence and HTA^[1]^ to inform its health benefit package for medicines, vaccines, and non-pharmacological interventions.^9-12^ Despite this, HTA has rarely been used to inform decisions made by Thailand’s Country Coordinating Mechanism (CCM). The CCM consists of multiple stakeholders representing local partners and acts as a governing body of GF programs in the country. Its status as a separate entity may have prevented the integration of the benefits package development mechanism into the CCM’s processes.

## The Value-for Money of Global Fund Programs: A Silver Lining

In 2012, the Thai CCM requested the Health Intervention and Technology Assessment Program (HITAP)^ [2]^ to conduct a mid-term review and evaluate the VFM of the GF’s program for HIV prevention targeting most-at-risk populations, namely, men that have sex with men (MSM), IDU, FSW, and migrant workers.^13^ While HTA^ [1]^ within the Thai health system has not been used specifically for infectious diseases within the population as a whole, its methods can still be used to understand the VFM of interventions for a targeted population. The results showed that the cost per infection averted is high and beyond the ceiling threshold (which at the time was 120 000 Thai baht per healthy life year gained), with the lowest cost for the IDU program at approximately 300 000 baht per HIV case averted and the highest for the MSM program at 11 million baht per case averted^ [3] ^. Three factors can explain the inefficiency of this program: firstly, targeting hard-to-reach marginal groups resulted in a higher cost; secondly, the focus on assisting non-formal health sector and community organizations, both of which do not have adequate infrastructure, in providing the services required significant capital investment and human resource training; and, thirdly, the program, having been implemented for only 2 years at the time of evaluation, had not yet reached its full potential due perhaps to lack of awareness and trust amongst the client or target population.

These three factors and the resulting inefficiency may be common in settings other than Thailand given that CCM managed GF programs separately from the country government; however, this does not mean that CCM should only support cost-effective interventions in their inception. An *ex-ante* evaluation should be performed before or during grant application and the *ex-post* evaluation routinely during the grant implementation.^14^ This is to ensure that the CCM can classify the interventions into categories shown in Figure. There are four possible types of interventions that are available for HIV, TB, and malaria. Intervention A shows good VFM from program commencement and in the long-term. Interventions B and C are cost-ineffective in the beginning but show improved VFM over time. The difference between interventions B and C is that the former can become cost-effective over a short period and even perhaps before a country’s graduation from GF support. Intervention D, on the other hand, is cost-ineffective from the beginning and even over time.

**Figure:**
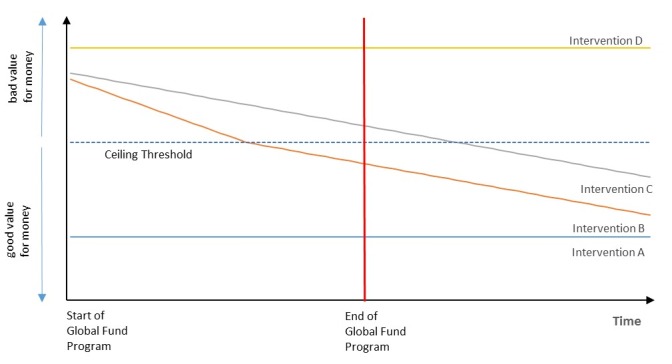
VFM of Possible Global Fund Programs/Interventions. Abbreviation: VFM, value-for-money.

In theory, country governments should fund interventions like intervention A without GF support; nevertheless, if there is insufficient health budget, then the GF should prioritize these types of interventions. It is difficult, however, to determine the differences between interventions B, C, and D in the beginning phase of their implementation. Investing in B, C, and D should be done carefully, ie, implementing those as pilots so that they can be easily discontinued if necessary before they are scaled up. Accordingly, GF and CCM should continuously include cost-effectiveness analysis in their monitoring and evaluation mechanisms to determine the VFM of these interventions (not just in terms of staying within budget, as is currently outlined in their previous funding model)^15^ and thus eventually detect and terminate the high-cost intervention D. As for interventions B and C, given that intervention B is well within the means of the government to continue implementation before or at the end of GF support, GF should promote prioritization of intervention B and countries’ corresponding ownership and responsibility for the program. Intervention C, which can eventually become cost-effective for the government post-graduation, can be considered as an optional investment for countries and a third priority.

The VFM can be monitored using both surrogate and final outcome. Observing the final outcome at the very beginning of program implementation is not often straightforward. For example, for a condom program, it is often not possible to monitor the population that uses a condom when having sex. As such, there is evidence of higher percentage of the population using condoms and the consequent reduction in HIV rate. However, at the end of the program, it will be necessary to measure the impact on HIV infections per se.

The following recommendations are offered to the GF, country governments and their CCMs to ensure the VFM for GF investment as well as the smooth transition from the GF grant once the countries become more self-sufficient.

## Policy Recommendations: To the Global Fund

- Continuing with ending one-size-fits-all governance approach for CCM – the GF needs to improve the understanding of the country context when working with them. With the New Funding Model in 2014-2016 and 2017-2019, the GF is moving in the right direction in increasing country dialogue and working with country governments’ national strategies.^15,16^ However, countries may have different health governance arrangements, and GF needs to keep certain principles and apply them based on the context. One example is the principle behind the CCM of having a multi-stakeholder process in designing and implementing GF programs. This is already available in the Thai context given that the National Health Security Office (NHSO) is responsible for treatment for HIV, TB, and malaria, and the prevention of HIV and TB already have multiple stakeholders on the NHSO board. If the GF works with the NHSO board, Thailand no longer requires a CCM and the benefit of using the existing governance structure is that it ensures country ownership, coordination, and integration of health policy, effective implementation and long-term sustainability.
- Making the best use of performance-based financing (PBF)^17^ – to avoid the perception of grants being a ‘windfall,’ the GF should emphasize PBF payments and ensure that the country is aware that if they are not performing well and keeping to the target, the grant disbursement will be deducted from the next round. With the GF’s introduction of the PBF, however, it needs to be careful in using the approach properly by avoiding punishing grant recipients who are working within their means but cannot reach their target, dubbed “poor performers,” because of other difficulties, eg, political conflict or war, disasters, other difficulties due to poor socio-economic status or target population, or factors beyond their control.
- Establishing an HTA^[1]^ mechanism for VFM framework – The GF needs to establish mechanisms and tools for the GF itself that will help CCM in countries to recognize whether the interventions submitted in the concept note or grant application are of category A, B, C, or D through *ex-ante* assessment, and be used for *ex-post* M&E of VFM of the GF program. In doing this, the GF may need to develop a technical manual, such as a reference case for economic evaluation and some standards of evidence to guide critical appraisal,^18^ and teams to assist countries for VFM assessments. The GF needs to compile data and information from grant applications and M&E processes that can in the future be used to guide GF and non-GF countries about the value of investment in HIV, TB and malaria interventions. In this case, GF or its assigned partner could act as an archivist and databank.
- Incorporating social and ethical dimensions in HTA for GF – to ensure the success of ending HIV, TB and malaria, the GF and country partners need to understand not only the health and economic impact of their investment but also the impact on equity of access to the GF programs. Understanding the social determinants of health on HIV, TB and malaria as well as their impact on programs targeting these diseases will be crucial to ensure that the most difficult-to-reach and at-risk populations can benefit from the program. As with the case in GF’s programs in Thailand, ending these diseases mean that even minority and outreach populations must be able to access these programs, and not just the majority of the affected population.^19,20^ Methods such as extended cost-effectiveness analysis or distributional cost-effectiveness analysis exist which can and have been applied to depict the distributional impact of allocation decisions and to help policy-makers make more equitable decisions.^21,22^
- The GF should use HTA for selection of health technologies and price negotiation for its central procurement, which can then assist other countries making procurements outside of the headquarters. This process will help drive VFM of the GF and avoid controversies in terms of questionable investments. Using VFM evidence for procurement of health technologies has advantages over price negotiations without using VFM evidence, which is the current practice in GF, due to its comparative advantage of bulk purchasing. Using VFM evidence for pricing means incentivizing industry to deliver good value (quite distinct from cheap or unprofitable) innovation. This also helps GF avoid the trap of investing in low-cost technologies that may have minimal impact or in engaging in a cost minimisation exercise, exerting its large purchasing power which countries are less able to do after transitioning and which may bring about complaints of stifling innovation.

Our recommendations are in line with donor performance agreements such as the UK’s Department for International Development (DfID)^23^ and with global funding conduits’ own VFM frameworks and strategies and are increasingly informing key performance indicators for such institutions. Indeed, the GF’s VFM and sustainability special initiative, often together with other stakeholders such as the World Bank, has encouraged the use of disease-specific models for optimising resource allocation such as in the case of Sudan where the HIV model Optima was used to inform the national strategic plan for HIV resulting in significant modelled improved health outcomes.^24^ Moreover, the GF Market Shaping Strategy makes an even clearer case for adopting cost-effectiveness analysis to inform product selection at central and country level, the latter through proactively funding in country cost-effectiveness analyses for its grantees: “The GF…will proactively engage with recipients to share relevant analyses and information about likely product costs and comparative health technology assessments…the GF Secretariat...will connect recipients with these resources to inform country-driven health technology assessment. Engaging in this process can also be an opportunity to build country capacity for health technology assessment and how to incorporate this into product selection decisions.”^25^

## Policy Recommendations: To the Countries

With support from GF and other international partners, the CCM (or equivalent authority) and local technical bodies need to develop capacity in using and assessing VFM as well as social, institutional, and ethical consequences of GF investments in the context of a broad HTA^[1]^ framework. The CCM must be able to work with local and international technical bodies that are supporting CCM and its work to continuously monitor and evaluate the VFM of their programs over time to classify interventions into A, B, C, or D. CCM has the authority to discontinue investments if they are found not to be good value for money. Moreover, the CCM and local partners need to understand the socio-economic, ethnic, and geographical factors that affect the programs’ effectiveness and to incorporate these concerns in their design and implementation. Using HTA in the decision-making process can also address these issues.

GF is appropriately geared to assist countries until they can sufficiently support themselves. However, during this period, countries should be able to engage with the GF work through a process that accounts for their existing public health programs and local challenges. Interventions funded through the GF should be a good value for investment, e.g. interventions A and B in Figure, for the country itself. Once the transition begins, the GF can re-invest the money saved from programs that are now transferred to the government to other important and/or targeted programs as well.

In conclusion, our paper illustrates the potential of aligning GF efforts with countries’ priority setting, ensuring that vertical programmes are considered alongside those under national health insurance schemes, which are broader and encompass many areas such as NCDs. It also demonstrates the utility of using economic evaluation to guide GF and CCM investment and management over time. This mechanism will ensure sustainability of cost-effective HIV, TB, and malaria interventions beyond the GF program and will ensure aid budgets, whilst they are relevant, go much further.^26^

## Acknowledgements

This case study is part of a larger work commissioned to the Results for Development (R4D) led by Dr. Robert Hecht and Arjun Vasan. The authors appreciate their comments and suggestions for the improvement of this case study.

The HITAP is funded by the Thailand Research Fund under the senior research scholar on Health Technology Assessment [Grant RTA59800011]. The HITAP International Unit (HIU) was established with support from the Thai Health-Global Link Initiative Project (TGLIP) and the international Decision Support Initiative (iDSI) to provide technical assistance on health intervention and technology assessment for governments of low- and middle-income countries. iDSI is funded by the Bill & Melinda Gates Foundation, the UK’s DfID, and the Rockefeller Foundation. This manuscript is partly supported by the iDSI [Grant OPP1087363]. The findings, interpretations, and conclusions expressed in this article do not necessarily reflect the views of the funding agencies.

## Ethical issues

Not applicable.

## Competing interests

Authors declare that they have no competing interests.

## Authors’ contributions

KK and YT conceptualized the ideas in the manuscript. KK, YT, KC, and AL contributed to the writing, production, and finalization of the manuscript. AL handled the administrative and submission process.

## Authors’ affiliations

^1^Bureau of Health Administration, Ministry of Public Health, Nonthaburi, Thailand. ^2^HITAP International Unit, Ministry of Public Health, Nonthaburi, Thailand. ^3^Global Health and Development Team, Imperial College London, London, UK. ^4^Health Intervention and Technology Assessment Program (HITAP), Ministry of Public Health, Nonthaburi, Thailand.

## Endnotes


[1] ‏ HTA refers to the systematic evaluation of properties, effects, and‏/or impacts of health technology. It is a multidisciplinary process‏ to evaluate the social, economic, organizational, and ethical issues of‏ a health intervention or health technology. The main purpose of‏ conducting an assessment is to inform a policy decision-making‏.

Health technology assessment. (n.d.). Retrieved August 18, 2015 from http://www.citationmachine.net/apa/cite-a-website?new=true‏.

[2] ‏ The HITAP is a semi-autonomous technical body under the Ministry of Public Health, Thailand, that uses HTA to inform UHC benefits package development in Thailand.

[3] The incremental cost-effectiveness ratios (ICERs) of the program were compared between the GF program and the Ministry of Public Health program. The ICER measures the cost of one healthy life year, accounting for the total program costs. The ceiling threshold is the government’s willingness-to-pay for one healthy life year.

